# Clozapine is a functional antagonist at cardiac human H_2_-histamine receptors

**DOI:** 10.1007/s00210-024-03683-7

**Published:** 2024-12-11

**Authors:** Jonas M. A. Schlicht, Undine Ahlrep, Britt Hofmann, Uwe Kirchhefer, Joachim Neumann, Ulrich Gergs

**Affiliations:** 1 Institute for Pharmacology and Toxicology, Medical Faculty, Martin-Luther-University Halle-Wittenberg, Magdeburger Straße 4, Halle (Saale), D-06097 Germany; 2 Department of Cardiac Surgery, Mid-German Heart Center, University Hospital Halle, Ernst Grube Straße 40, Halle (Saale), D-06097 Germany; 3 Institute for Pharmacology and Toxicology, University Münster, Domagkstraße 12, Münster, D-48149 Germany

**Keywords:** Clozapine, H_2_-histamine receptor, Heart, Inotropy, Chronotropy

## Abstract

Clozapine is an atypical antipsychotic (neuroleptic) drug. Clozapine binds to H_2_-histamine receptors in vitro. We wanted to test the hypothesis that clozapine might be a functional antagonist at human cardiac H_2_-histamine receptors. To that end, we studied isolated electrically stimulated left atrial preparations and spontaneously beating right atrial preparations from transgenic mice with cardiomyocyte-specific overexpression of the human H_2_-histamine receptor (H_2_-TG). For comparison, we used wild-type littermate mice (WT). Finally, we measured isometric force of contraction in isolated electrically stimulated muscle strips from the human right atrium (HAP) obtained from patients during bypass surgery. After pre-stimulation with histamine, clozapine (up to 10 µM) concentration and time dependently decreased beating rate in right atrial preparations from H_2_-TG. Clozapine concentration dependently 1, 3, and 10 µM decreased histamine-stimulated force of contraction in HAP. Clozapine (10 µM) decreased also the isoprenaline-stimulated force of contraction in HAP. In summary, clozapine can antagonize the function of H_2_-histamine and β-receptors in the human heart.

## Introduction

**Fig. 1:**
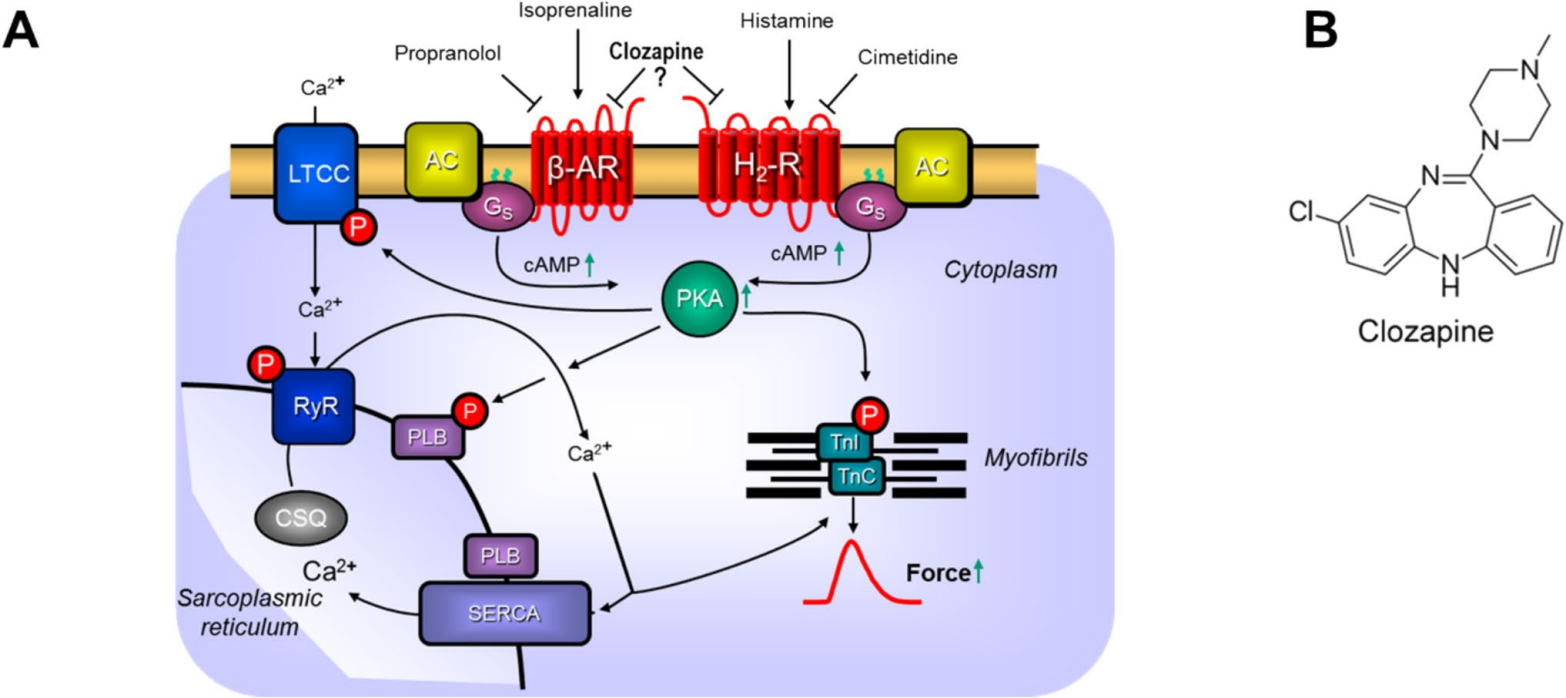
Mechanism of action of H_2_-histamine receptor in cardiomyocytes and structural formula. **A** Scheme of hypothetical signal transduction of H_2_-histamine receptors and β-adrenoceptors in human atrial cardiomyocytes. Stimulation of H_2_-histamine receptors by histamine or stimulation of β-adrenoceptors by isoprenaline lead to the production of cAMP. The increase of cAMP leads to an increase in force of contraction. Cimetidine inhibits the human H_2_-histamine receptor. **B** Structural formula of clozapine

Clozapine (Fig. 1B), a derivative of imipramine, was developed as an antidepressant agent in the year 1959 (Crilly 2007). Clozapine was widely studied for other indications than depression in psychiatry in the 1960s (Crilly 2007). Clozapine turned out to be an antipsychotic drug (Crilly 2007). Clozapine has the advantage compared to haloperidol and similar antipsychotic drugs that clozapine less often induced extrapyramidal side effects (Crilly 2007). This is explained by the fact that clozapine in contrast to haloperidol or chlorpromazine binds less to D_2_-dopamine receptors in the brain (Crilly 2007). Clozapine blocks not only some dopamine receptors but also other receptors or ion channels in the central nervous system and peripheral organs (Siafis et al. 2023, Yeh et al. 2023). For instance, clozapine is also an atropine-like antagonist at human muscarinic receptors (Crilly 2007). This is invoked to explain the tachycardia seen in some patients on treatment with clozapine (Nilsson et al. 2018). Moreover, clozapine blocks vascular α-adrenoceptors, which can explain the hypotension in some patients treated with clozapine (Crilly 2007). The antipsychotic effects of clozapine may result from an antagonistic effect in the brain at some dopamine receptors (most potently at D_4_-dopamine receptors) but also 5-HT_2A_-serotonin receptors (Khokhar et al. 2018). A clinical problem with clozapine is that it can lead to lethal agranulocytosis, possibly by interaction with H_4_-histamine receptors (Goto et al. 2016, de Leon et al. 2020). Clozapine can lead under chronic therapy to several cardiac side effects notably myocarditis (Ronaldson 2017, de Leon et al. 2020). In vitro, clozapine inhibited currents stimulated by β-adrenoceptor agonists like isoprenaline on L-type calcium currents (LTCC) in rat cardiomyocytes (Zhao et al. 1997). Clozapine binds to human β-adrenoceptors with an inhibitory constant (*K*_i_) of 5000 nM (Roth and Driscol 2024). In spite of its many untoward effects, clozapine is still used as a second choice if side effects of drugs like haloperidol are not tolerated or if haloperidol-like drugs are found to be ineffective to treat the psychiatric disease (Pattnaik et al. 2023). Hence, it is still relevant to understand the cardiac effects of clozapine in the human heart better and avoid thus untoward cardiac side effects.

In the present context, it is important to know that clozapine is also an antagonist at H_1_-histamine receptors (pK_i_-value, negative decadic logarithm of the inhibition constant calculated from radioligand competition binding assays: 8.58) and H_2_-histamine receptors (pK_i_-value = 6.28) in ligand binding and biochemical signal transduction studies (Appl et al. 2012). The therapeutic drug concentration for clozapine lies around 1 µM (Appl et al. 2012). Thence, binding of clozapine to histamine receptors could occur in the clinic.

The cardiac side effects of clozapine are well studied and can include deadly arrhythmias. Clozapine can block potassium channels leading to long QT syndromes and a possible increased cardiac mortality (Titier et al. 2005).

Histamine increases force of contraction via H_2_-histamine receptors in many species (Panula et al. 2015, Neumann et al. 2021). However, species differences and regional differences exist in the cardiac effects of histamine (Neumann et al. 2021). In humans, H_2_-histamine receptors were measurable in both the atrium and ventricle (radioligand binding: Baumann et al. 1982, 1983, 1984; antibody and mRNA expression: Matsuda et al. 2004). In humans, the cardiac H_2_-histamine receptors mediate a positive inotropic effect in isolated human atrial cardiac preparations (Fig. 1A). The mechanisms involve stimulation of cAMP generation and subsequent activation of cAMP-dependent protein kinase (Fig. 1A). This kinase phosphorylates several substrates that lead to an increase in the rate of force development and a reduction of the time of relaxation (Levi et al. 1981, Genovese et al. 1988, Zerkowski et al. 1993, Thoren et al. 2011, Sanders et al. 1996). To obtain an animal model for the histamine action in the human heart, we have generated transgenic mice that overexpress human H_2_ receptors in the heart (Gergs et al. 2019). In these mice (H_2_-TG), histamine exerted in left atrial preparations (LA) a positive inotropic effect, and in right atrial preparation (RA), histamine exerted a positive chronotropic effect, whereas positive inotropic effects of histamine were missing in littermate wild-type mice (Gergs et al. 2019). To the best of our knowledge, the functional effects of clozapine on H_2_-histamine receptors in the human heart have not yet been reported. To test the clinical relevance of our initial findings in mice, we set out to measure the effects of clozapine under isometric conditions on the force of contraction in the human heart. To this end, we used electrically stimulated right atrial strips obtained rapidly from patients suffering from severe coronary heart disease.

In summary, we studied the following hypotheses: firstly, clozapine blocks H_2_-histamine receptor-stimulated contractility in H_2_-TG. Secondly, clozapine reduces histamine-stimulated contractility in the isolated human atrium via H_2_-histamine receptors.

## Methods

### Transgenic mice

The investigation conforms to the Guide for the Care and Use of Laboratory Animals published by the National Research Council (2011). Animals were maintained and handled according to approved protocols of the animal welfare committees of the University of Halle-Wittenberg, Germany. The generation and initial characterization of the transgenic mice (H_2_-TG) have been described before (Gergs et al. 2010, 2019). In brief, for generation of transgenic mice by pronuclear DNA injection, human H_2_-receptor cDNA was inserted into a mouse cardiac α-myosin heavy chain promoter expression cassette. For all experiments, adult transgenic mice and WT littermates of both sexes were used.

### Contractile studies in mice

As described before, the right or left atrial preparations from the mice were isolated and mounted in organ baths (Gergs et al. 2013; Neumann et al. 1998). The bathing solution of the organ baths contained (in mM) the following: 119.8 NaCI, 5.4 KCI, 1.8 CaCl_2_, 1.05 MgCl_2_, 0.42 NaH_2_PO_4_, 22.6 NaHCO_3_, 0.05 Na_2_EDTA, 0.28 ascorbic acid, and 5.05 glucose. The solution was continuously gassed with 95% O_2_ and 5% CO_2_ and maintained at 37 °C and pH 7.4 (Neumann et al. 1998, Kirchhefer et al. 2004). Spontaneously beating right atrial preparations from mice were used to study any chronotropic effects, and left atrial preparations were used to study any ionotropic effects. After equilibration was reached, histamine or isoprenaline was cumulatively added to left atrial or right atrial preparations to establish concentration-response curves. Then, 1, 3, and 10 µM clozapine were cumulatively added to the organ bath. In separate experiments, concentration-response curves to isoprenaline in mouse left and right atrial preparations were obtained either after pre-incubation with 10 µM clozapine or alone.

### Contractile studies on human preparations

The contractile studies on human preparations used the same setup and buffer as in the mouse studies. The samples were obtained from 13 male patients and 2 female patients, 52–87 years old. Our methods used for atrial contraction studies in human samples have been previously published and were not altered in this study (Gergs et al. 2009; Gergs et al. 2021).

### Data analysis

Data shown are means ± SEM. Statistical significance was estimated by analysis of variance followed by Bonferroni’s *t*-test and/or Student’s *t*-test as indicated in the legends. A *p*-value < 0.05 was considered significant.

### Drugs and materials

Clozapine, cimetidine, isoprenaline, histamine, and propranolol were purchased from Sigma-Aldrich (Taufkirchen, Germany). All other chemicals were of analytical grade. Demineralized water was used throughout the experiments. Stock solutions were freshly prepared daily.

## Results

### Left atrium from H_2_-TG

**Fig. 2:**
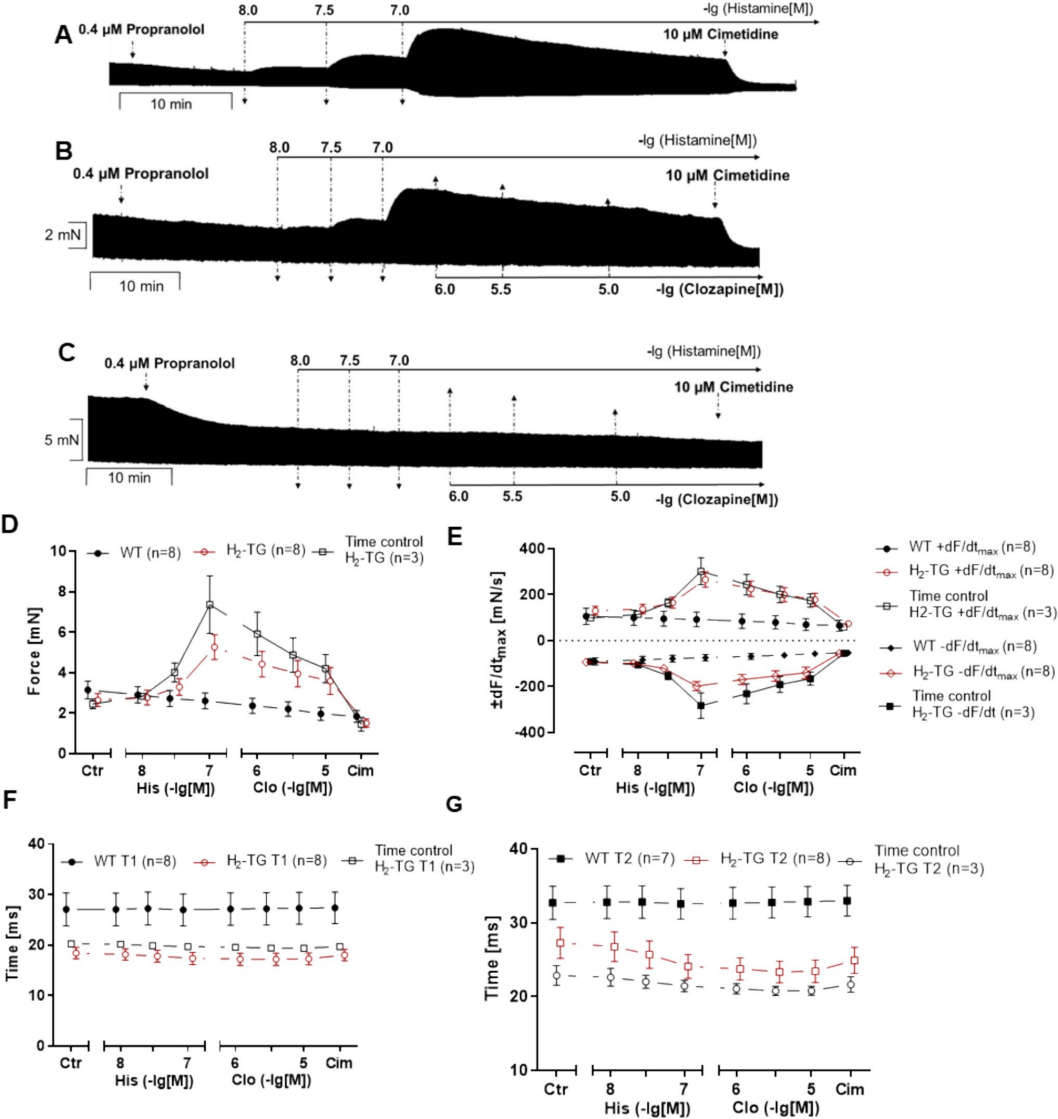
Effects of clozapine on left atria of H2-TG in the presence of histamine. **A** Original recording of the time-dependent effect of 100 nM histamine and in the presence of 0.4 µM propranolol (time control). **B**, **C** Original recording of the concentration- and time-dependent effects of 1, 3, and 10 µM clozapine followed by cimetidine in H_2_-TG mice (**B**) and wild-type mice (**C**) after histamine stimulation and pre-incubation with propranolol. Summary of effects of 1, 3, and 10 µM clozapine (Clo) by 10 µM cimetidine (Cim) on mouse left atrial preparations in the presence of 100 nM histamine (His). **C** Force of contraction. Force (Ctr in WT) = 3.15 ± 0.43 mN and force (Ctr in H_2_-TG) = 2.66 ± 0.32 mN. Force of time control (Ctr in H_2_-TG) = 2.46 ± 0.24 mN. **D** Maximum rate of tension development (dF/dt_max_) and maximum rate of relaxation (−dF/dt_max_) in mN/s: +dF/dt_max_ (Ctr in WT) = 106.20 ± 35.75, +dF/dt_max_ (Ctr in H_2_-TG) = 129.81 ± 19.27, +dF/dt_max_ of time control (Ctr in H_2_-TG) = 99.05 ± 9.25; −dF/dt_max_ (Ctr in WT) = −90.27 ± 16.09, −dF/dt_max_ (Ctr in H_2_-TG) = −93.56 ± 11.28, −dF/dt_max_ in time control (Ctr in H_2_-TG) = −90.76 ± 7.55. **E** Time to peak tension (T1) and time to relaxation (T2) in milliseconds (ms): T1 (Ctr in WT) = 27.08 ± 3.27, T1 (Ctr in H_2_-TG) = 18.43 ± 1.13, T1 of time control (Ctr in H_2_-TG) = 20.27 ± 0.371; T2 (Ctr in WT) = 29.59 ± 3.18, T2 (Ctr in H_2_-TG) = 29.37 ± 2.88, T2 of time control (Ctr in H_2_-TG) = 22.85 ± 1.34. The force of contraction before addition of histamine and in the presence of propranolol was designated the control value (Ctr). Number in brackets indicates number of experiments. Comparison between WT mice (closed circle) and H_2_-TG mice with (open circle) and without (open square) the addition of clozapine. Abscissae indicate concentrations of histamine and clozapine in negative decadic molar concentrations

We started with experiments in H_2_-TG to provide proof of concept that clozapine can interact with human cardiac H_2_-histamine receptors. We first applied propranolol to rule out the stimulatory effects of endogenous noradrenaline on β-adrenoceptors (0.4 µM). Subsequently, histamine was additionally and cumulatively applied. In left atrial preparations from H_2_-TG (Fig. 2A, B, left-hand side) but not from WT (left-hand side, Fig. 2C), histamine exhibited a time- and concentration-dependent positive inotropic effect, in agreement with our initial study (Gergs et al. 2019). In WT, neither histamine nor clozapine had a positive inotropic effect (Fig. 2C). These data are summarized with regard to the force of contraction for H_2_-TG and WT in Fig. 2D.

Furthermore, histamine also augmented the absolute values of the rate of tension development and the rate of relaxation (Fig. 2E). Moreover, in the same samples, histamine shortened time to peak tension and the time of relaxation (Fig. 2F). When subsequently clozapine was added, we noted that the positive inotropic effects of histamine were attenuated (Fig. 2B). The remaining positive inotropic effects of histamine were weakened by additional cimetidine that is a selective H_2_ antagonist. This shows that clozapine can attenuate the positive inotropic effect (PIE) of histamine on human H_2_ receptors in principle. Moreover, clozapine is less effective to reduced FOC than cimetidine at H_2_ receptors. In addition, the experiments with cimetidine confirm the histamine-induced PIE is really H_2_-histamine receptor-mediated. The PIE of histamine is present in H_2_-TG but not WT. The negative inotropic effect (NIE) of clozapine is evident after histamine application in H_2_-TG (Fig. 2A, B, C). Moreover, the increase in the rate of contraction and the rate of relaxation by histamine is antagonized by clozapine (Fig. 2E). Likewise, the shortening of time to peak tension or time of relaxation by histamine was reversed by subsequently applied clozapine in H_2_-TG (Fig. 2F, G).

### Right atrium from H_2_-TG

**Fig. 3:**
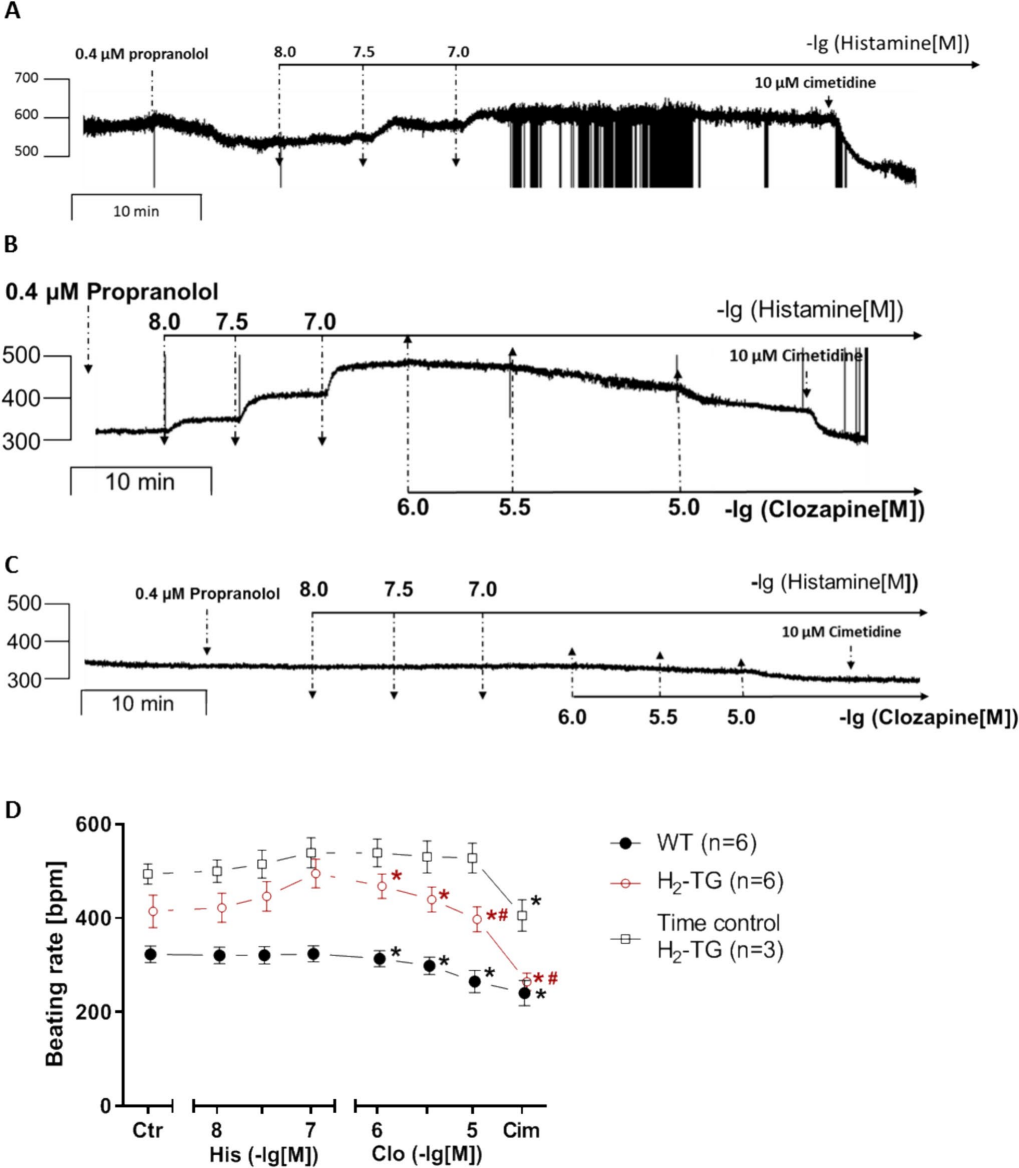
Effects of clozapine on right atria of H_2_-TG in the presence of histamine. **A** Original recordings of the time-dependent effects of 100 nM histamine and in the presence of 0.4 µM propranolol (time control). **B**, **C** Original recordings of the concentration- and time-dependent negative chronotropic effects of clozapine in H_2_-TG (**B**) and WT (**C**). **D** Effects of 1, 3, and 10 µM clozapine (Clo) followed by 10 µM cimetidine (Cim) on beating rate of mouse right atria in the presence of 100 nM histamine (His). The beating rate before addition of histamine and in the presence of propranolol was designated the control value (Ctr). Beating rate in beats per minute (bpm): Beating rate (Ctr in WT) = 323.18 ± 17.48, beating rate (Ctr in H_2_-TG) = 414.72 ± 34.50, and beating rate time control (Ctr in H_2_-TG) = 494.17 ± 21.44. Number in brackets indicates number of experiments. Comparison between WT mice (closed circle) and H_2_-TG mice with (open circle) and without (open square) the addition of clozapine. Abscissae indicate concentrations of histamine and clozapine in negative decadic molar concentrations. *p < 0.05 vs. 100 nM histamine. #*p* < 0.05 vs. time control

Next, we were interested in right atrial function, under the same experimental conditions used in the left atrium. Histamine time and concentration dependently increased the beating rate of right atrial preparations from H_2_-TG but not from WT, as already published (Gergs et al. 2019). The beating rate was displayed in an original muscle strip in Fig. 3A, B (H_2_-TG), as well as Fig. 3C (WT). Summarized data for the beating rates can be found in Fig. 3D (H_2_-TG and WT). Please note that histamine is more potent to raise the beating rate than force (cf. Fig. 2D). We suggest that therefore clozapine was more potent and effective to reduce the beating rate than force. In the right atrium, we again finally applied cimetidine. As elaborated above, cimetidine was more effective than clozapine to reduce histamine-stimulated beating rate. This indicates firstly that histamine acted via H_2_ receptors and secondly that these specific effects were antagonized by clozapine.

### Left atrium from wild type

**Fig. 4:**
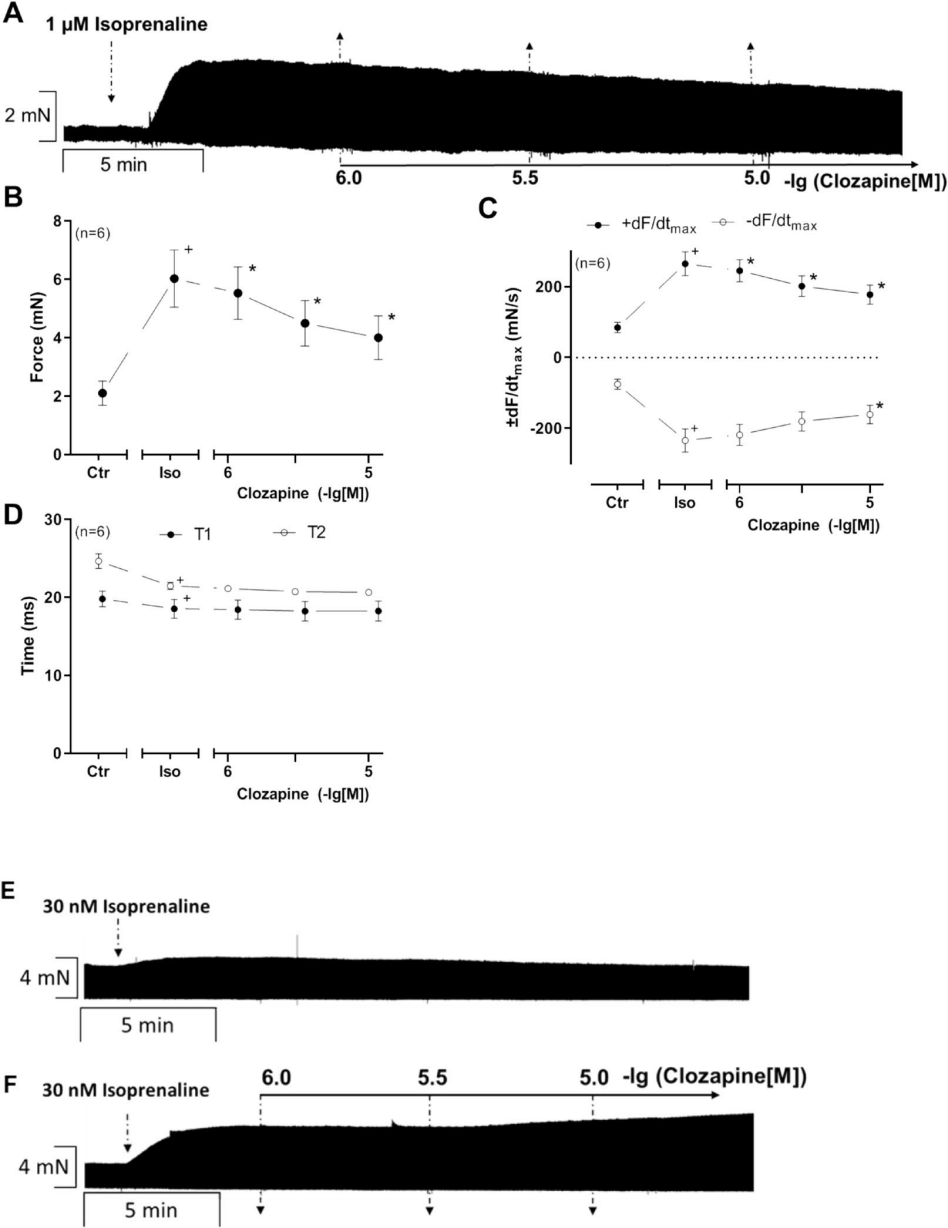

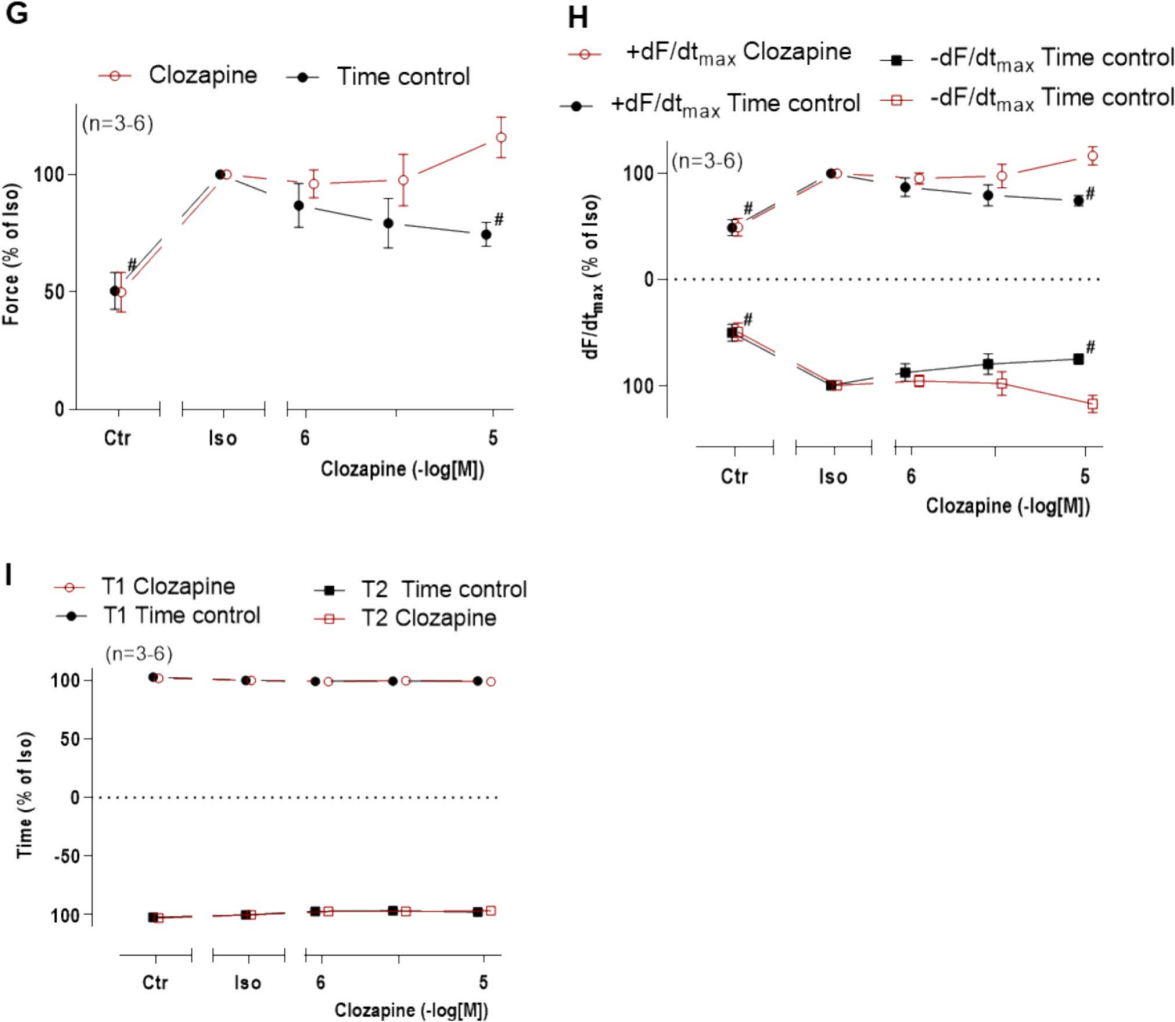
Effects of clozapine on left atria of WT in the presence of isoprenaline. **A** Original recording of the concentration- and time-dependent effects of 1, 3, and 10 µM clozapine on WT mouse right atria after 1 µM isoprenaline (Iso). **B** Force of contraction. Force (Ctr in WT) = 2.11 ± 0.42 mN. **C** Maximum rate of tension development (dF/dt_max_) and maximum rate of relaxation (−dF/dt_max_) in mN/s: +dF/dt_max_ (Ctr in WT) = 84.84 ± 14.91; −dF/dt_max_ (Ctr in WT) = −75.29 ± 14.08. **D** Time to peak tension (T1) and relaxation (T2) in ms: T1 (Ctr in WT) = 19.80 ± 1.02; T2 (Ctr in WT) = 24.67 ± 0.94. **E** Original recording of the time-dependent effects of 30 nM isoprenaline (time control). **F** Original recording of the time- and concentration-dependent effects of 1, 3, and 10 µM clozapine in the presence of 30 nM isoprenaline. **G** Force of contraction. Force (Ctr in WT) = 1.93 ± 0.46 mN, force time control (Ctr in WT) = 2.57 ± 0.67. **H** Maximum rate of tension development (dF/dt_max_) and maximum rate of relaxation (−dF/dt_max_) in mN/s: +dF/dt_max_ (Ctr in WT) = 72.79 ± 21.00, +dF/dt_max_ time control (Ctr in WT) = 105.66 ± 31.07; −dF/dt_max_ (Ctr in WT) = −53.69 ± 11.33, −dF/dt_max_ time control (Ctr in WT) = −77.64 ± 19.71. **I** Time to peak tension (T1) and relaxation (T2) in ms: T1 (Ctr in WT) = 25.93 ± 3.74, T1 time control (Ctr in WT) = 25.21 ± 3.29; T2 (Ctr in WT) = 36.45 ± 8.25, T2 time control (Ctr in WT) = 35.05 ± 6.80. Number in brackets indicates number of experiments. The force before addition of isoprenaline was defined as the control value (Ctr). Where indicated the force in the presence of 30 nM isoprenaline was defined as 100%. Abscissae indicate concentrations of clozapine in negative decadic molar concentrations. +*p* < 0.05 vs. Ctr; *p <0.05 vs. 1 µM isoprenaline, #*p* < 0.05 vs. 30 nM isoprenaline

As mentioned in the “Introduction” section, there is published evidence in rat cardiomyocytes that clozapine can antagonize the electrophysiological effects of β-adrenoceptor stimulation (Zhao et al. 1997). Hence, we stimulated with LA with 1 µM isoprenaline to raise FOC. Subsequently, clozapine was additionally and cumulatively added. Here, clozapine exhibited a time- and concentration-dependent negative inotropic effect as seen in the original recording (Fig. 4A). These data are summarized with regard to the force of contraction (Fig. 4B), rate of tension development and relaxation (Fig. 4C), and time to peak tension and relaxation (Fig. 4D). Here in the left atrium from WT, isoprenaline increased force, rate of relaxation, and rate of tension development and shortened time to peak tension and time of relaxation. The contractile effects of 1 µM isoprenaline (which was maximum effective) were reversed by clozapine. We speculated whether in the presence of a lower concentration of isoprenaline, clozapine would still be antagonistic. This was done because under clinical conditions, a submaximal stimulation of β-adrenoceptor might occur more frequently than a maximum stimulation with 1 µM isoprenaline. However, at lower concentrations of isoprenaline (30 nM), we observed an opposite effect. Here, clozapine even stimulated force of contraction. We speculate that this might mean that clozapine is a partial agonist at β-adrenoceptors. This is seen in an original recording in Fig. 4F and should be compared with a time control in Fig. 4E. These data are summarized in Fig. 4G for force of contraction, rate of tension development, and rate of relaxation in Fig. 4H and on time to peak tension and time of relaxation in Fig. 4I.

**Fig. 5:**
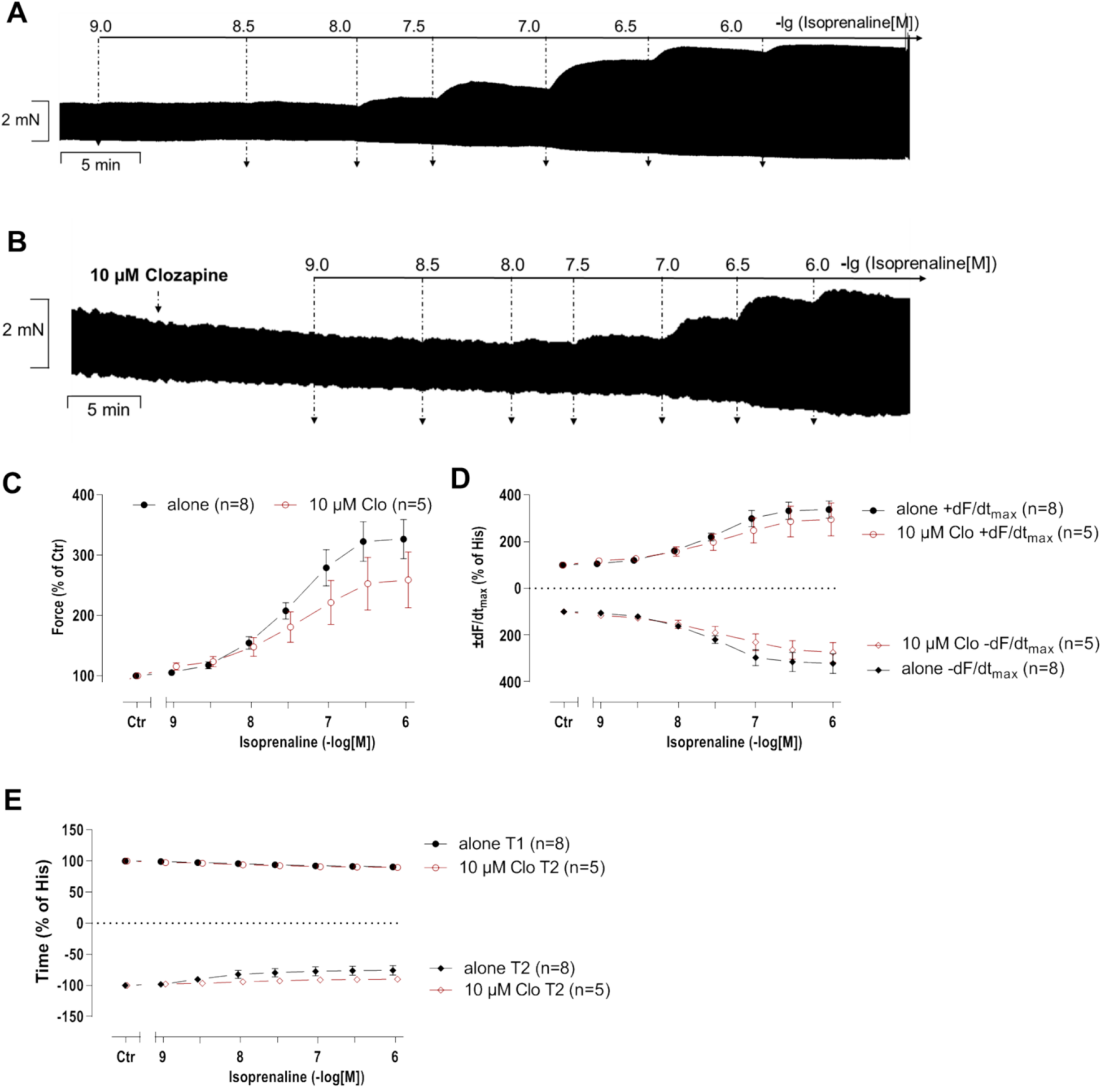
Effect of isoprenaline on left atria of WT in presence of clozapine. Concentration- and time-dependent effects of isoprenaline (Iso) on WT mouse left atria in the absence (alone) or presence of 10 µM clozapine (Clo). The force before the addition of isoprenaline and where indicated in the presence of clozapine were designated as the control values (Ctr) and set to 100%. Numbers in brackets indicate number of experiments. Abscissae indicate concentrations of isoprenaline in negative decadic molar concentrations. Original recording of isoprenaline alone (**A**) and in the presence of clozapine (**B**). **C** Force of contraction. Force (100%) = 3.32 ± 0.11 mN. **D** Maximum rate of tension development (dF/dt_max_ (100%) = 140.84 ± 10.7 mN/s) and maximum rate of relaxation (*−*dF/dt_max_ (100%) = *−*101.57 ± 13.32 mN/s). **E** Time to peak tension and relaxation. T1 (100%) = 22.91 ± 1.03 ms. T2 (100%) = 37.61 ± 6.76 ms

In separate experiments, isoprenaline (1 nM to 1 µM) was cumulatively applied in absence (Fig. 5A) or presence (Fig. 5B) of 10 µM clozapine. In the presence of clozapine, the maximum force of contraction induced by isoprenaline appeared to be reduced, although this effect did not gain statistical significance (Fig. 5C). Similarly, the maximum rate of contraction and relaxation was reduced in the mean values but this reached not significance (Fig. 5D). Finally, a summary of the effect on time to peak tension and relaxation is shown in Fig. 5E. There was clearly no effect of clozapine on time to peak tension, but clozapine diminished albeit not significantly time of relaxation. Hence, effects of clozapine on force parameters in mice were minor if relevant at all.

### Right atrium from wild-type mice

**Fig. 6:**
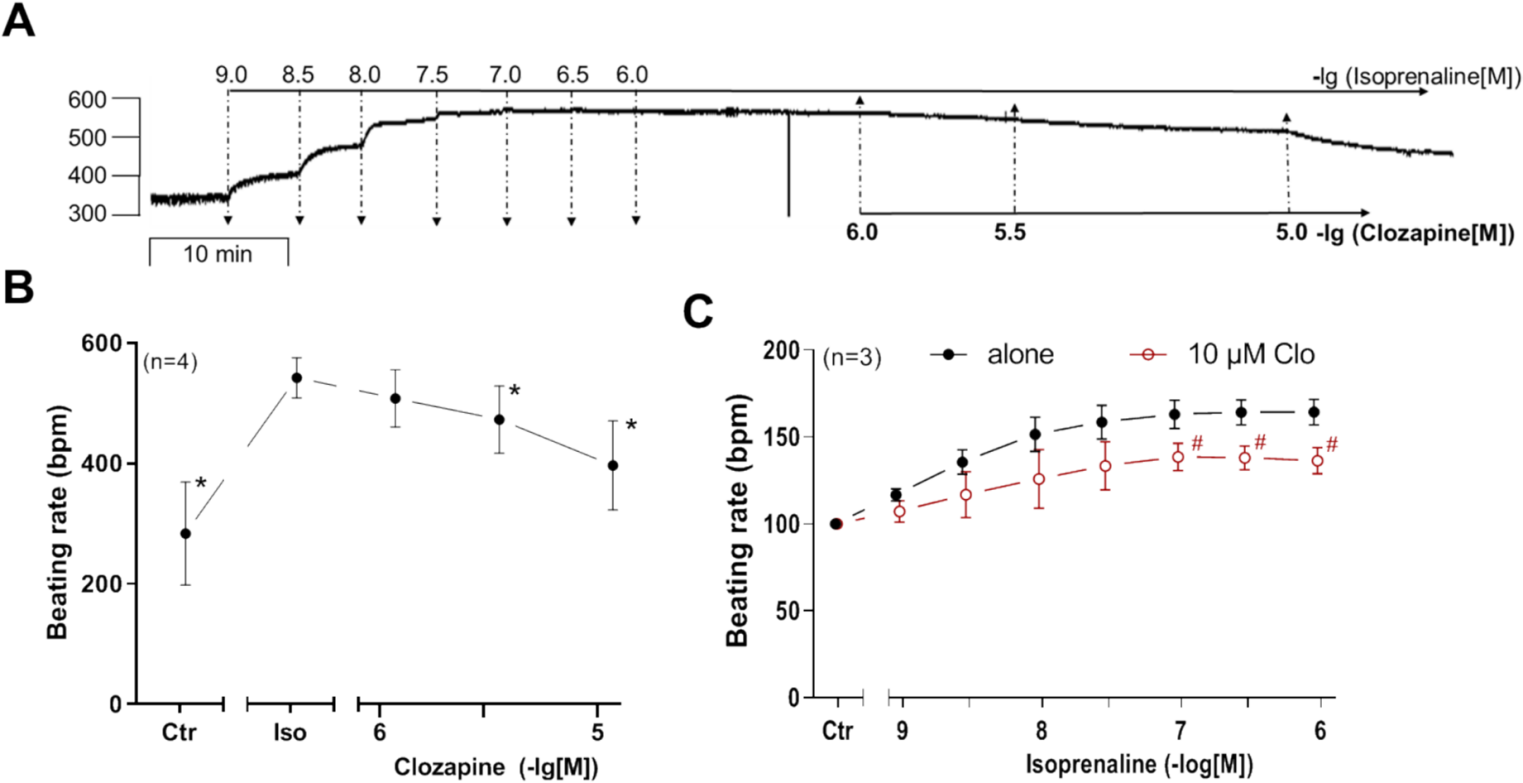

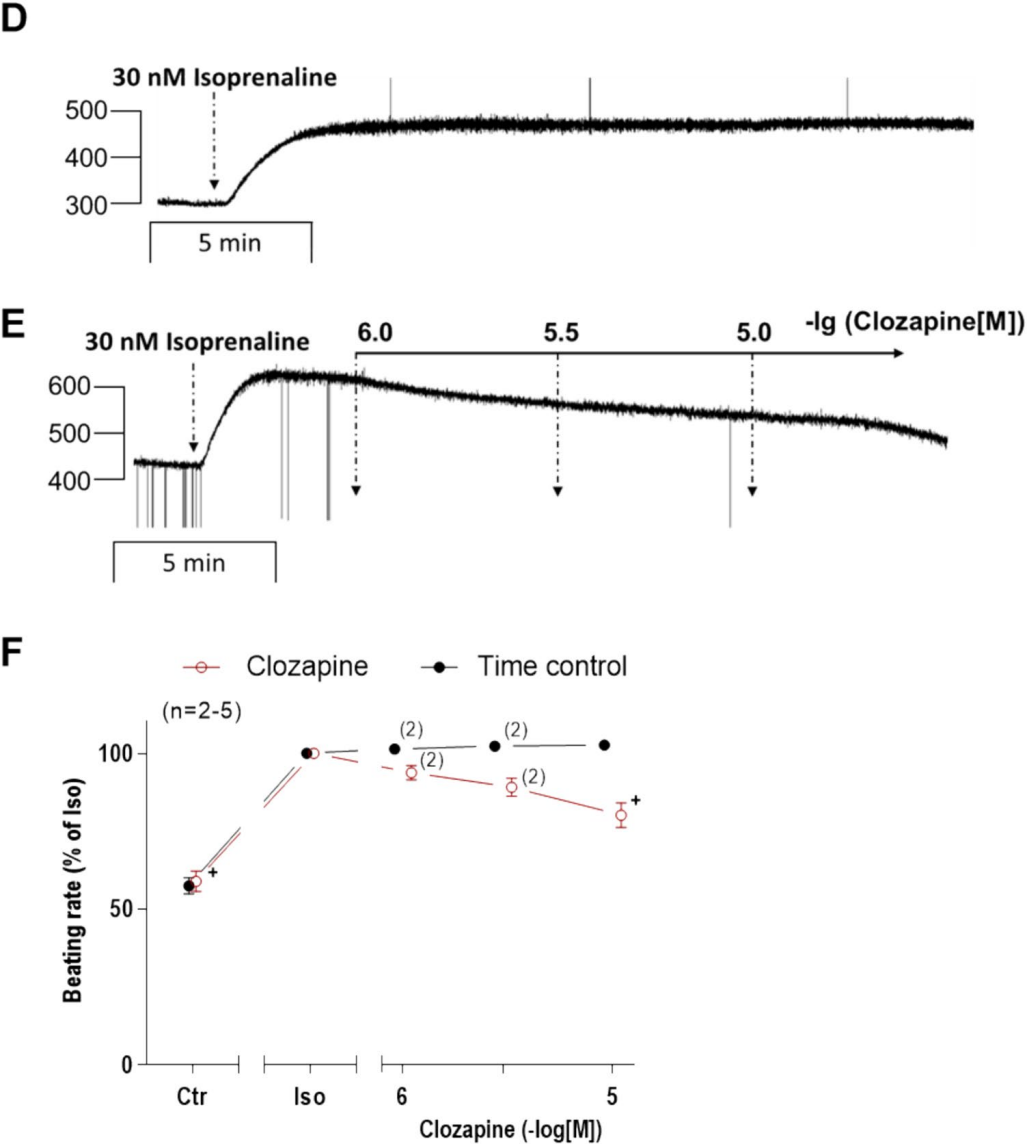
Effect of clozapine in combination with isoprenaline on right atria of WT. Original recording (**A**) and summary (**B**) of the concentration- and time-dependent effects of clozapine on beating rate in beating wild-type mice right atria (WT). **C** Effects of isoprenaline on WT mouse right atria in the absence (alone) or presence of 10 µM clozapine (Clo). The beating rate before addition of isoprenaline and where indicated in the presence of clozapine was designated to the control value (Ctr) and set to 100%. Beating rate (100%) = 288.85 ± 17.91 bpm. **D** Original recording of time-dependent effects of 30 nM isoprenaline on beating rate (time control). **E** Original recording of the time- and concentration-dependent effects of 1, 3, 10 µM clozapine in the presence of 30 nM isoprenaline. **F** Summary of the time- and concentration-dependent effects of 1, 3, and 10 µM clozapine (open circle) in comparison to time control (closed circle) in WT. Beating rate (BPM) in the presence of 30 nM isoprenaline was designated the control value and set to 100%. Beating rate (100%) = 318.56 ± 58.94 bpm, beating rate time control (100%) = 280.24 ± 31.47 bpm. *p < 0.05 vs. 1 µM isoprenaline (Iso). #*p* < 0.05 vs. WT. +*p* < 0.05 vs. 30 nM isoprenaline (Iso). Numbers in brackets indicate number of experiments. Abscissae indicate concentrations of clozapine and isoprenaline in negative decadic molar concentrations

Next, we were interested in right atrial function, under the same experimental conditions used in the left atrium. Isoprenaline concentration dependently increased the beating rate of right atrial preparations from WT. Clozapine exhibits a negative chronotropic effect (NCE) when applied in succession to isoprenaline (Fig. 6A, B). The presence of 10 µM clozapine before addition of isoprenaline partly inhibited the positive chronotropic effect (PCE) of isoprenaline (Fig. 6C). For comparison with the force values in Fig. 4F, we decided to test whether clozapine might act as a partial agonist at lower positive chronotropic concentrations of isoprenaline (30 nM) which are not maximum (cf. Fig. 5C). In contrast to the findings in Fig. 4F, clozapine did not increase beating rate but rather reduced beating rate. This is seen in an original recording in Fig. 6E. Please note in Fig. 6D that these effects cannot be explained by a run down over time. Several such data are summarized in Fig. 6F, showing that the anti-β-adrenergic negative chronotropic effect of 10 µM clozapine was significant.

### Effects in the human atrium

**Fig. 7:**
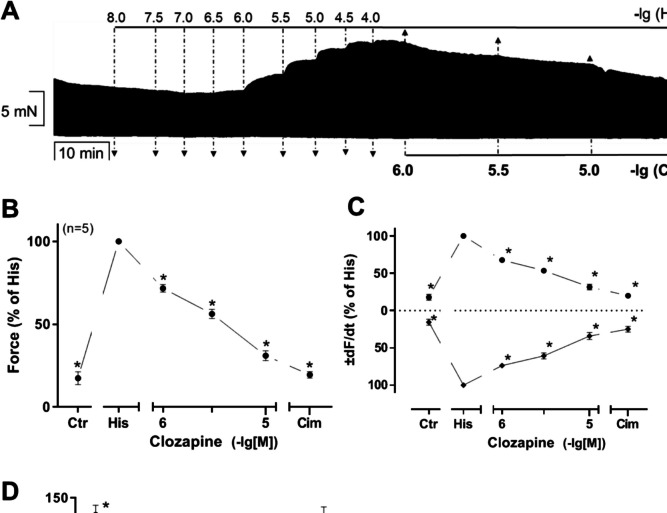
Effect of clozapine in HAP in the presence of histamine. Time-dependent effects of 1, 3, and 10 µM clozapine followed by 10 µM cimetidine in the presence of 100 µM histamine in HAP. The force of contraction before the addition of clozapine and in the presence of histamine was defined as the control (100%). *p < 0.05 vs. 100 µM histamine. Number in brackets indicates number of experiments. Abscissae indicate concentrations of histamine and clozapine in negative decadic molar concentrations. **A** Original recording of the time-dependent negative inotropic effect of clozapine. **B** Force of contraction. Force (100%) = 8.95 ± 4.29 mN. **C** Maximum rate of contraction and relaxation. dF/dt_max_ (100%) = 218.16 ± 57.89 mN/s. *−*dF/dt_max_ (100%) = *−*91.72 ± 8.68 mN/s. **D** Time to relaxation. T2 (100%) = 108.29 ± 10.84 ms

Firstly, concentration-response curves for histamine (100 nM to 100 mM) were established. Afterwards, clozapine (1, 3, 10 µM) was additionally and cumulatively applied. Histamine exerted a positive inotropic effect, which was partly antagonized by clozapine as can be seen in an original recording (Fig. 7A). As a control, we added 10 µM cimetidine which was able to reverse any remaining PIE of histamine (Fig. 7A). As reported before by us and others, histamine in HAP increased force of contraction (Fig. 7B), the maximum rate of contraction and relaxation (Fig. 7C), and histamine reduced the time to peak tension and relaxation (Fig. 7D). Additionally, applied clozapine concentration and time dependently reduced these histamine-induced augmentations in force of contraction as seen in an original recording (Fig. 7A) and summarized in Fig. 7B. Likewise, the maximum rate of contraction and relaxation was reduced by additionally added clozapine (Fig. 7C).

**Fig. 8:**
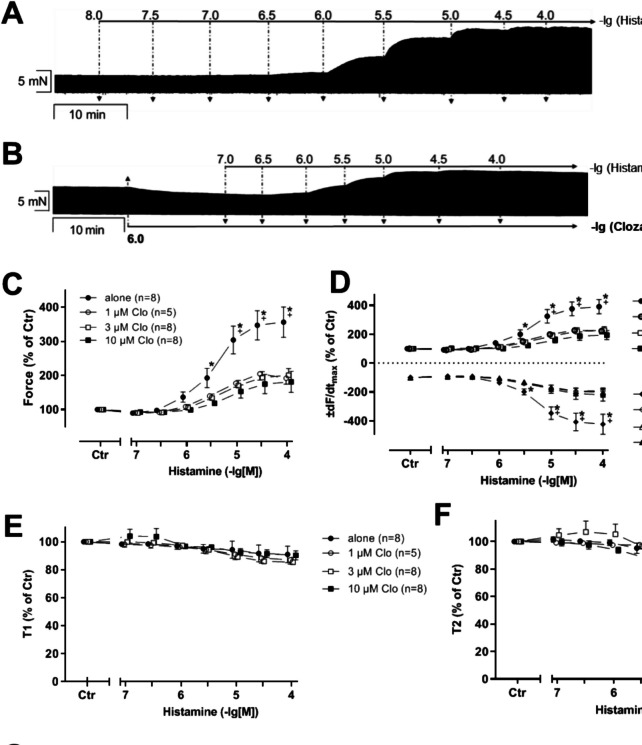
Effect of histamine in HAP in the presence of clozapine. Concentration- and time-dependent effects of histamine (His) in human atrial preparations in the absence (alone) or presence of 1, 3, or 10 µM clozapine (Clo). The effects before addition of histamine and where indicated in the presence of clozapine were designated to the control value (Ctr) and set to 100%. Numbers in brackets indicate number of experiments. Abscissae indicate concentrations of histamine in negative decadic molar concentrations. Original recording of histamine alone (**A**) and in the presence of 1 µM clozapine (**B**). Negative inotropic effect of 1 µM clozapine (**B**) in HAP. **C** Force of contraction. Force (100%) = 6.68 ± 1.39 mN. *p < 0.05 vs. 10 µM Clo. +*p* < 0.05 vs. 1 and 3 µM Clo. **D** Maximum rate of contraction and relaxation. dF/dt_max_ (100%) = 89.56 ± 11.26 mN/s. −dF/dt_max_ (100%) = −46.71 ± 6.95 mN/s. **E** Time to peak tension. T1 (100%) = 56.50 ± 3.26 ms. **F** Time to relaxation. T2 (100%) = 141.88 ± 9.15 ms. **G** Effect of 1 µM clozapine on force of contraction in HAP. #*p* < 0.05 vs. Tyrode’s solution (Ctr). Force (100%) = 3.35 ± 0.76 mN

In separate experiments, histamine (100 nM to 100 µM) was cumulatively applied in the absence (Fig. 8A) or presence (Fig. 8B) of clozapine. In the presence of clozapine, the maximum force of contraction induced by histamine was reduced significantly (Fig. 8C). The maximum rate of contraction (Fig. 8D) was affected in a similar way. The time of relaxation (Fig. 8F) was reduced by histamine, and this effect was attenuated by pre-incubation with clozapine. There may be a similar tendency in the time to peak tension. However, quantitatively, these changes were too small to reach significance (Fig. 8E). As seen in an original recording in Fig. 8B, clozapine (1 µM) alone exerts a negative inotropic effect, which is summarized in Fig. 8F.

**Fig. 9:**
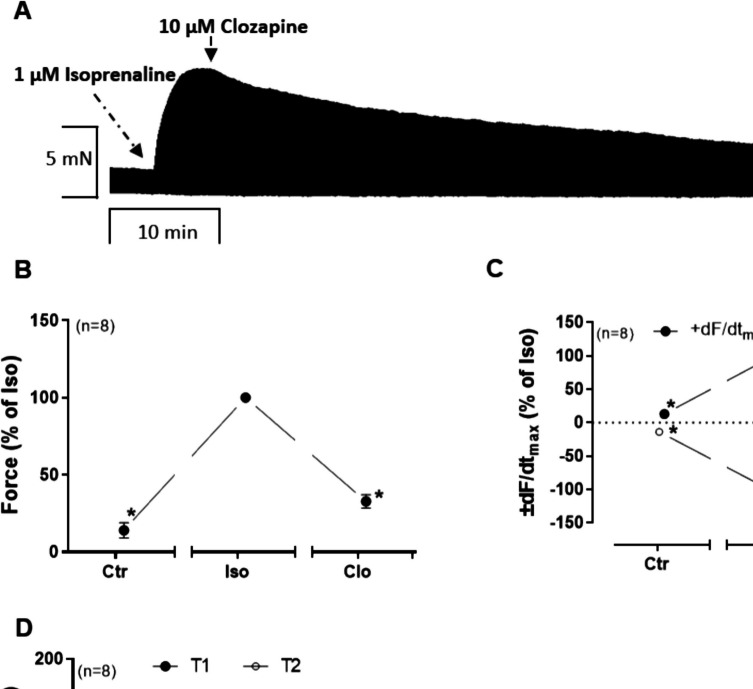
Effect of clozapine in HAP in the presence of histamine. Time-dependent effects of 10 µM clozapine (Clo) in the presence of 1 µM isoprenaline (Iso) in HAP. The force of contraction before the addition of clozapine and in the presence of isoprenaline was designated to the control value (Ctr) and set to 100%. *p < 0.05 vs. 1 µM isoprenaline. Number in brackets indicates number of experiments. **A** Original recording of the time-dependent negative inotropic effect of clozapine. **B** Force of contraction. Force (100%) = 8.06 ± 2.37 mN. **C** Maximum rate of contraction and relaxation. dF/dt_max_ (100%) = 145.35 ± 35.87 mN/s. −dF/dt_max_ (100%) = −85.09 ± 19.71 mN/s. **D** Time to peak tension and relaxation. T1 (100%) = 45.01 ± 2.93 ms. T2 (100%) = 92.56 ± 6.98 ms

In order to compare β-anti-adrenergic effects which we noted at least with regard to the beating rate in mice (Fig. 6B, F), we chose also to perform similar experiments in HAP. The question arose whether with respect to FOC clozapine might reduce isoprenaline-stimulated FOC in HAP. This was the case. We first applied 1 µM isoprenaline to maximally raise FOC. This was followed by 10 µM clozapine in HAP (Fig. 9A). The force of contraction was increased by isoprenaline, while subsequent clozapine addition lowered force of contraction (Fig. 9B). The maximum rate of contraction and relaxation changed accordingly (Fig. 9C). Time to peak tension and relaxation were not altered by additionally applied clozapine (Fig. 9D).

## Discussion

The main new findings are that clozapine has a negative inotropic effect alone or after histamine stimulation in HAP. In addition, negative inotropic effects after isoprenaline stimulation in HAP were observed.

We confirmed that histamine in our HAP exerted a PIE by stimulation of H_2_-histamine receptors because the inotropic effect of histamine was reversed by cimetidine. These histamine-induced positive inotropic effects were reduced by clozapine in HAP. In a different approach, also pre-incubation with clozapine attenuated the subsequent positive inotropic effect of histamine. These two different approaches are consistent with the view that clozapine is functionally antagonistic at H_2_-histamine receptors in HAP. The data suggests that clozapine acts as a non-competitive antagonist at H_2_-histamine receptors by reducing the maximum force of contraction under full receptor stimulation. Published data concur that H_2_-histamine receptor stimulation in HAP leads to cAMP increases and activation of cAMP-dependent protein kinase, phosphorylation of regulatory proteins, and this leads to the positive inotropic and relaxant effects of histamine in HAP (Fig. 1, review: Neumann et al. 2021).

Clozapine also antagonizes the H_1_-histamine receptors (Roegge et al. 2007, Humbert-Claude et al. 2012), and this might explain the weight gain under clozapine treatment (Hong et al. 2002). Whether H_1_-histamine receptors are functional in the HAP is understudied. One cannot exclude an effect of clozapine on human cardiac H_1_-histamine receptors, but this needs to be subject for further study. We have shown that in transgenic mice with overexpression of histamine H_1_-receptors, histamine can increase FOC (Rayo Abella et al. 2024). Hence, one might predict that clozapine can block histamine receptors in the heart of H_1_-TG but also in histamine H_1_-receptor stimulation in HAP. Clozapine antagonizes at H_2_-histamine receptors (Appl et al. 2012, IC50 value = 12 µM: Humbert-Claude et al. 2012). The antipsychotic effects of clozapine may be mediated in part by the inhibition of central H_4_- and/or H_3_-histamine receptors (Alves-Rodrigues et al. 1996, Humbert-Claude et al. 2012). In the rat heart, clozapine inhibited isoprenaline-stimulated currents through the L-type calcium ion channel (LTCC, Zhao et al. 1997).

### Clinical relevance

Therapeutic plasma concentrations of clozapine range from 0.3 to 2.4 µM (Jann et al. 1993, Appl et al. 2012, Ronaldson 2017). Hence, the cardiac effect we noted here might exist in patients. Interestingly, clozapine treatment is correlated with an increased incidence of heart failure (Clapham et al. 2023), possibly by interactions at histamine H_2_ receptors but also with β-adrenoceptors. At least in the brain of animals chronically (but not acutely) treated with clozapine, the expression of subunits of the cAMP-dependent protein kinase was altered (Dwivedi et al. 2002). In this way in patients chronically treated with clozapine, the activity of cAMP-dependent protein kinases in the heart may be altered. But this needs to be elucidated. Clozapine can induce cardiomyopathies possibly by detrimental effects on cardiac mitochondria (Zhang et al. 2024). Our data might argue that acutely given clozapine might decrease force of contraction by antagonizing H_2_- and β-receptors both of which increase force of contraction in humans.

However, the clinical situations where histamine in contrast to noradrenaline plays a role in maintaining force of contraction may be rather rare. Therefore, in the clinical setting, a cardiac role of clozapine might be also rare. Nevertheless, one should be very careful and use a low dosage in psychiatric patients with known heart failure when one gives clozapine.

### Limitations

 We have not had the opportunity to study human sinus nodes or human ventricular samples in our contraction study due to a lack of access to these tissues.

In conclusion, we show that clozapine is a functional antagonist at H_2_-histamine receptors in the human heart.

## Data Availability

All source data for this work (or generated in this study) are available upon reasonable request.
